# *Thinopyrum* species as a genetic resource: enhancing salt tolerance in wheat and forage crops for sustainable agriculture

**DOI:** 10.3389/fpls.2025.1728305

**Published:** 2025-12-17

**Authors:** Wei Li, Hongwei Li, Qi Zheng

**Affiliations:** 1 State Key Laboratory of Seed Innovation, Institute of Genetics and Developmental Biology, Chinese Academy of Sciences, Beijing, China; 2 College of Agriculture, Yangtze University, Jingzhou, China

**Keywords:** *Thinopyrum*, salt tolerance, distant hybridization, transcriptomics, proteomics, salt-responsive genes

## Abstract

*Thinopyrum* species are native to coastal regions and have evolved notable salt tolerance mechanisms, including efficient Na^+^ exclusion and K^+^ retention, enhanced antioxidant capacity, and the accumulation of compatible solutes for osmoregulation. Among the *Thinopyrum* species, *Th. ponticum* has long been used as saline pasture and energy plant, which was recently suggested for the construction of a “Coastal Grass Belt” around the Bohai Sea. The salt tolerance in some *Thinopyrum* species, such as *Th. ponticum*, *Th. elongatum*, *Th. bessarabicum*, and *Th. distichum* have been transferred into wheat as (partial) amphiploid, addition, substitution, translocation, and introgression lines. The introgression lines with enhanced salt tolerance, derived from wheat × *Th. ponticum* had been utilized as salt-tolerant wheat varieties. In addition, amphiploids and perennial wheat have been developed as salt-tolerant forage crops. Salt tolerance in *Thinopyrum* species is governed by multiple genes, which have been mapped principally to homologous chromosomes group 3 and group 5. Transcriptomic and proteomic analyses have revealed a number of differentially expressed genes (proteins) involved in the salt tolerance response in *Thinopyrum* species; however, few of these have been functionally characterized. Therefore, further work is needed to investigate gene networks underlying salt tolerance in *Thinopyrum*, which may serve as molecular targets for the genetic improvement of salt-tolerant forage crops such as *Tritipyrum* and staple crops like wheat.

## 1 Introduction

Genus *Thinopyrum* Á. Löve, erected in 1980 (Löve, 1980), consists of the species with E or J (= E^b^) genome which have been classified as *Triticum*, *Agropyron*, *Elymus*, *Elytrigia*, and *Lophopyrum* previously. For sake of consistency with the existing literature, this paper uses *Thinopyrum*, notwithstanding the recent adoption of *Lophopyrum* by Yen and Yang (2022). *Thinopyrum* species include diploids (2n=14) *Th. elongatum* (Host) D.R. Dewey and *Th. bessarabicum* (Săvul. and Rayss) Á. Löve, tetraploids (2n=28) *Th. corsicum* (Hack.) Banfi, *Th. curvifolium* (Lange) D.R. Dewey, *Th. distichum* (Thunb.) Á. Löve, *Th. flaccidifolium* (Boiss. and Heldr.) Moustaka, *Th. junceiforme* (Á. Löve and D. Löve) Á. Löve, *Th. sartorii* (Boiss. and Heldr.) Á. Löve, and *Th. elongatum* (Host) D.R. Dewey, hexaploids (2n=42) *Th. junceum* (L.) Á. Löve and *Th. intermedium* (Host) Barkworth and D.R. Dewey, one octoploid (2n=56) *Th. turcicum* (P.E. McGuire) B.R. Baum, and one decaploid (2n=70) *Th. ponticum* (Podp.) Z.W. Liu and R.C. Wang. The detail information of *Thinopyrum* species is listed in **Table 1**. Most *Thinopyrum* species are maritime grasses native to the shores of the Baltic, Mediterranean, and North Sea, whereas *Th. distichum* originates from the coastal Cape Provinces of South Africa (Pienaar et al. 1988). As wild relatives of wheat, *Thinopyrum* species plays a pivotal role in wheat genetic improvement for resistance to biotic and abiotic stresses, including salinity (King et al., 1997a; Colmer et al., 2006; Urbanavičiūtė et al., 2021; Korostyleva et al., 2023; Tounsi et al., 2024).

**Table 1:** *Thinopyrum* species list documented for salt tolerance.

Thinopyrum species	Genome	Homotypic synonyms (from POWO)	Salt tolerance	References
*Th. elongatum* (Host) D.R. Dewey	J^e^J^e^ (2n=14)	*Agropyron elongatum*, *Agropyron rigidum*, *Elymus elongatus*, *Elytrigia elongata*, *Lophopyrum elongatum*, *Triticum elongatum*.	90% survival rate @ 500 mM NaCl	McGuire and Dvôrák (1981)
*Th. bessarabicum* (Săvul & Rayss) Á. Löve	J^b^J^b^ (2n=14)	*Agropyron bessarabicum*, *Agropyron junceum*, *Elytrigia bessarabica*, *Elymus farctus*, *Elytrigia juncea*, *Lophopyrum bessarabicum*.	90% survival rate @ 350 mM NaCl; K^+^/Na^+^ = 1.41 @ 200 mM NaCl	Gorham et al. (1985); Gorham et al. (1986a)
*Th. corsicum* (Hack.) Banfi	J_1_J_1_J_1_J_1_ (2n=28)	*Agropyron caespitosum*, *Agropyron corsicum*, *Agropyron elongatum*, *Elymus corsicus*, *Elymus nodosus*, *Elytrigia corsica*, *Lophopyrum corsicum*.	Not available	
*Th. curvifolium* (Lange) D.R. Dewey	J^b^J^b^J^b^J^b^ (2n=28)	*Agropyron curvifolium*, *Elymus curvifolius*, *Elytrigia curvifolia*, *Lophopyrum curvifolium*, *Pauneroa curvifolia*, *Triticum curvifolium*.	20% survival rate @ 500 mM NaCl	McGuire and Dvôrák (1981)
*Th. distichum* (Thunb.) Á. Löve	J^b^J^b^J^e^J^e^ (2n=28)	*Agropyron distichum*, *Elymus distichus*, *Elytrigia disticha*, *Triticum distichum*.	44%−83% survival rate @ 500 mM NaCl	McGuire and Dvôrák (1981)
*Th. elongatum* (Host) D.R. Dewey	J^e^J^e^J^e^J^e^ (2n=28)	*Agropyron scirpeum*, *Agropyron elongatum*, *Elytrigia scirpea*, *Lophopyrum scirpeum*, *Th. scirpeum*	87% survival rate @ 750 mM NaCl; K^+^/Na^+^ = 1.32 @ 250 mM NaCl	McGuire and Dvôrák (1981); Gorham et al. (1986a)
*Th. flaccidifolium* (Boiss. & Heldr.) Moustaka	J_1_J_1_J_2_J_2_ (2n=28)	*Agropyron elongatum*, *Agropyron scirpeum*, *Elymus elongatus*, *Elymus flaccidifolius*, *Elytrigia flaccidifolia*, *Lophopyrum flaccidifolium*.	Not available	
*Th. junceiforme* (Löve & Löve) Á. Löve	J^b^J^b^J^e^J^e^ (2n=28)	*Agropyron junceiforme*, *Agropyron junceum*, *Elymus farctus*, *Elymus junceiformis*, *Elytrigia juncea*, *Elytrigia junceiformis.*	89% survival rate @ 750 mM NaCl; K^+^/Na^+^ = 0.99−1.56 @ 200 mM NaCl	McGuire and Dvôrák (1981); Gorham et al. (1986a)
*Th. sartorii* (Boiss. & Heldr.) Á. Löve	J^b^J^b^J^e^J^e^ (2n=28)	*Agropyron junceum* var. *sartorii*, *Th. junceum*.	K^+^/Na^+^ = 0.43 @ 250 mM NaCl	Gorham et al. (1986a)
*Th. intermedium* (Host) Barkworth & D.R. Dewey	J^vs^J^vs^J^r^J^r^StSt (2n=42)	*Agropyron glaucum*, *Agropyron intermedium*, *Elytrigia intermedia*, *Trichopyrum intermedium*, *Triticum intermedium*, *Triticum repens*.	5%−17% survival rate @ 750 mM NaCl	McGuire and Dvôrák (1981)
*Th. junceum* (L.) Á. Löve	J_1_J_1_J_2_J_2_EE (2n=42)	*Agropyron junceum*, *Agropyron repens*, *Braconotia juncea*, *Elymus multinodus*, *Elytrigia juncea*, *Festuca juncea*, *Frumentum junceum*, *Triticum junceum*.	K^+^/Na^+^ = 1.02 @ 200 mM NaCl	Gorham et al. (1986a)
*Th. turcicum* (P.E. McGuire) B.R. Baum & D.A. Johnson	(2n=56)	*Elytrigia turcica*, *Lophopyrum turcicum*.	40% survival rate @ 500 mM NaCl	McGuire and Dvôrák (1981)
*Th. ponticum* (Podp.) Barkworth & D.R. Dewey	JJJJJJJ_s_J_s_J_s_J_s_ (2n=70)	*Agropyron elongatum*, *Elymus elongatus*, *Elymus ponticus*, *Elytrigia elongata*, *Elytrigia pontica*, *Lophopyrum ponticum*, *Triticum ponticum*.	12%−100% survival rate @ 750 mM NaCl	Shannon (1978); McGuire and Dvôrák (1981)

POWO (Plants of the World Online), https://powo.science.kew.org/.

Salt-affected soils cover approximately 932 million hectares (ha) of land worldwide, accounting for more than 10% of cropland (Rengasamy, 2006). This situation is further aggravated with faulty irrigation practices (Mohanavelu et al., 2021). Soil salinity is generally expressed as EC_e_ (the electrical conductivity of saturated paste extract), EC_1:5_ (the electrical conductivity of soil:water = 1:5), the percentage of total soluble salts relative to soil (w/w), and exchangeable sodium percentag (ESP). Na^+^ exclusion is considered a major mechanism of salt tolerance in plants. Low Na^+^ accumulation and high K^+^/Na^+^ ratio in shoots are generally used as indices to discriminate salt-tolerant plants. To meet the ever-increasing food demand of the global human population, it is essential to extend the cultivation of food and forage crops in salt-affected soils. Salt stress usually inhibits photosynthesis, water and nutrient absorption, and plant growth, ultimately resulting in premature senescence and yield loss. Therefore, developing salt-tolerant varieties is a priority to maximize crop productivity and adaptability in saline-alkaline soils. *Thinopyrum* species have acquired high levels of salt tolerance from the coastal saline-alkaline environments. The transfer of salt tolerance from *Thinopyrum* into wheat has been thoroughly reviewed (King et al., 1997a; Colmer et al., 2006; Urbanavičiūtė et al., 2021; Kotula et al., 2024). This paper summarized recent approaches for investigating salt tolerance, salt-responsive genes, and pathways in *Thinopyrum* species.

## 2 Salt tolerance across *Thinopyrum* species

### 2.1 Salt tolerance in *Th. ponticum*

*Th. ponticum*, commonly known as tall wheatgrass, has long been cultivated as saline pasture and energy plant as well as for soil reclamation (Andrioli, 2023). Up till now, more than ten *Th. ponticum* cultivars have been released since the 1950s (Li et al., 2022a, b). A number of documents demonstrated that *Th. ponticum* is among the most salt-tolerant forage crops (Pearson and Bernstein, 1958; Dewey, 1960; Greenway and Rogers, 1963; Rogers and Bailey, 1963; Mcguire and Dvôrák, 1981; Roundy, 1983; Johnson, 1991; Pearen et al., 1997; Shen et al., 1999; Peng et al., 2002; Shen et al., 2003; Huang et al., 2006; Huang and Liang, 2007; Suyama et al., 2007a, 2007; Meng et al., 2009; Sepehry et al., 2012; Temel et al., 2015; Riedell, 2016; Borrajo et al., 2022; Zhang et al., 2022a; Xiao et al., 2025). For instance, several accessions of *Th. ponticum* showed high survival rates in the stepwise-increased final concentration of 750 mM NaCl (Shannon, 1978; McGuire and Dvôrák, 1981) and maintained reasonable growth in saline soil with EC_e_ = 13.9 dS m^–1^ (Dewey, 1960). The 50% inhibition of germination and seedling emergence rates of two varieties, Tyrrell and Dundas, occurred at 300 and 110 mM NaCl, respectively (Zhang et al., 2005). *Th. ponticum* grew well and produced 6820, 5230, and 2920 kg ha^–1^ dry matter yield in high saline (EC_e_ = 9.80 dS m^–1^, pH = 8.5, ESP = 11.9), high alkali (EC_e_ = 0.89 dS m^–1^, pH = 10.3, ESP = 60.5), and high saline-alkali soils (EC_e_ = 9.08 dS m^–1^, pH = 9.4, ESP = 49.7), respectively (Temel et al., 2015). However, according to 50% inhibition of shoot biomass, the salt tolerance of *Th. ponticum* was sometimes lower than that of alfalfa (*Medicago sativa*) (Sagers et al., 2017). Additionally, it has a low survival rate and does not grow well in very high-saline soils (EC_e_ > 100 dS m^–1^) (Semple et al., 2008). Under saline/waterlogged conditions, puccinellia (*Puccinellia ciliata* Bor. cv. Menemen) grows better than *Th. ponticum* (Jenkins et al., 2010). A surface soil salinity of less than 1% (w/w) is recommended for the cultivation of *Th. ponticum* in coastal saline land, allowing for a high survival rate (Asay and Jensen, 1996; Li et al., 2023a). Once irrigation with saline water having EC_w_ ≤ 5.42 dS m^−1^ and SAR ≤ 36.31 in late spring was recently suggested for tall wheatgrass production in the “Coastal Grass Belt” targeted area (Li et al., 2023c), which resulted in minimal risk of soil salinization after rainfall leaching in summer.

The salt tolerance in *Th. ponticum* has been transferred into wheat as partial amphiploids and translocation/introgression lines. For instance, the BC_1_ and BC_2_ offspring from *Triticum aestivum* × *Th. ponticum* can survive and set seeds under 350 mM NaCl stress for 30 days (Dvořák et al., 1985). The CS-*Th. ponticum* amphiploid, which has 54 chromosomes plus a pair of telosomes, accumulated less Na^+^ in the shoots than CS when exposed to 275 mM NaCl (Schachtman et al., 1989). A few salt-tolerant wheat introgression lines were established through somatic hybridization between *T. aestivum* cv. Jinan 177 and *Th. ponticum* (Wang et al., 2004), among which Shanrong 3 produced 22.6% higher grain yield than Dekang 961, a local salt-tolerant wheat cultivar, in soils with a salinity level of 0.3%−0.5% (Shan et al., 2006). Additionally, the salt tolerance of Shanrong 3 was manifested by high rates of both seed germination and seedling survival under 340 mM NaCl stress (Liu et al., 2012). Another introgression line, Shanrong 4, exhibited enhanced alkalinity tolerance under 100 mM mixed salt stress (NaHCO_3_: Na_2_CO_3_ = 9: 1, pH = 8.9) (Meng et al., 2017). In addition, a wheat-*Th. ponticum* translocation line, S148, was able to germinate in 400 mM NaCl solution (Yuan and Tomita, 2015). Recently, the durum wheat*-Th. ponticum* 7AL•7el_1_L recombinant lines with enhanced salt tolerance were developed (Tounsi et al., 2024).

### 2.2 Salt tolerance in the diploids *Th. elongatum* and *Th. bessarabicum*

*Th. elongatum* can survive under gradually increased 500 mM NaCl but cannot grow in 750 mM NaCl (McGuire and Dvôrák, 1981). An amphiploid from *T. aestivum* cv. Chinese Spring (CS) × *Th. elongatum*, which has complete *Th. elongatum* genome, exhibited a higher survival rate and produced more dry matter and seed yields than CS when exposed to 250 mM NaCl, 250 mM KCl, 75 mM MgSO_4_, 150 mM K_2_SO_4_, and 18.0 or 36.0 g L^–1^ of marine salt stress in hydroponic tanks (Dvořák and Ross, 1986) and in saline soil (Omielan et al., 1991). Under 200 mM NaCl stress, the CS-*Th. elongatum* amphiploid transports less Na^+^ into the shoots than CS (Schachtman et al., 1989). Another CS-*Th. elongatum* amphiploid line, Y1805, may synthesize more proline and soluble sugars and have higher activities of superoxide dismutase and catalase than CS under salt stress (Peng et al., 2022). The durum wheat-*Th. elongatum* 7EL recombinant lines greatly mitigated the effects of salt stress on root and leaf growth and enhanced the accumulation of photosynthetic pigments, compatible solutes, and antioxidant like ascorbate (Tounsi et al., 2024). *Th. bessarabicum* can withstand prolonged exposure to 350 mM NaCl in hydroponic culture and was considered an osmoconformer (Gorham et al., 1985). The CS-*Th. bessarabicum* amphiploid showed a higher survival rate and grain yield than CS in 250 mM NaCl (Gorham et al., 1986b; Forster et al., 1987; King et al., 1997b). Several *Tritipyrum* lines with enhanced salt tolerance and seed productivity have been developed from the offspring of tetraploid wheat *T. durum* × *Th. bessarabicum* (King et al., 1997b), which have the potential to be used as a new type of forage crop. In addition, perennial wheat derived from *Thinopyrum* also provides a great opportunity for both food and forage crops with enhanced salt tolerance (Cui et al., 2018).

### 2.3 Salt tolerance in other *Thinopyrum* species

The salt tolerance also exists in other polyploid *Thinopyrum* species (McGuire and Dvôrák, 1981; Rozema et al., 1983; Gorham et al., 1986a). For instance, both *Th. junceiforme* and *Th. scirpeum* can survive in stepwise-increased concentrations of 750 mM NaCl. *Th. distichum* showed a survival rate of 44%−83% in 500 mM NaCl, which is lower than that of *Th. elongatum* (McGuire and Dvôrák, 1981). One octoploid, *Th. turcicum*, previously described as *Elyt. turcica* (McGuire, 1983) and recently recognized as a distinct *Thinopyrum* species (Baum and Johnson, 2017), had a survival rate of 40% under 500 mM NaCl stress (McGuire and Dvôrák, 1981). The ratios of K^+^/Na^+^ increased according to *Th. sartorii* (0.43), *Th. junceum* (1.02), *Th. scirpeum* (1.32), and *Th. bessarabicum* (1.41) when cultured in 250 mM NaCl (Gorham et al., 1986a). The ratios of K^+^/Na^+^ in four accessions of *Th. junceiforme* ranged between 0.99 and 1.56 under 250 mM NaCl stress, indicating that genetic variation in salt tolerance exists among the *Th. junceiforme* genotypes (Gorham et al., 1986a). *Th. junceiforme*, commonly known as sea wheatgrass, is a segmental allotetraploid species (Genome J_1_J_1_J_2_J_2_, Dewey, 1984). Recently, a *T. turgidum* (emmer wheat)-sea wheatgrass amphiploid line, 13G819, germinated better than the emmer wheat parent under 200 mM NaCl stress (Li et al., 2019).

The CS-*Th. elongatum* amphiploid produced more yield and had a higher ratio of K^+^/Na^+^ than CS, the CS-*Th. bessarabicum* amphiploid, and the CS-*Th. scirpeum* amphiploid under 100−150 mM NaCl stress (Akhtar et al., 1994). The *CS-Th. elongatum* amphiploid was more tolerant and transported less Na^+^ from roots to shoots than the CS-*Th. scirpeum* amphiploid under 200 mM NaCl stress (Gorham, 1994). The salt tolerance of the durum wheat-*Th. bessarabicum* amphiploids was stronger than that of the durum wheat-*Th. distichum* amphiploids (Marais et al., 2014). Notably, *Th. ponticum* (acc. PI 276399) grew faster and had stronger selectivity for K^+^ over Na^+^ than the tetraploid *Elyt. elongata* (acc. PI 578686) in the presence of 100–200 mM NaCl (Guo et al., 2015). Furthermore, salt tolerance increased according to AgCS (CS×*Th. elongatum-*unkn.acc., genome ABDE), CSLt (CS×*Th. turcicum*, genome ABDEEEE), and LDNLp (durum wheat-LDN×*Th. ponticum*, genome ABEEEEE), indicating that the salt tolerance of the amphipods is dependent on the number of *Thinopyrum* chromosomes (Abbasi et al., 2020). Therefore, salt-tolerant crops may be developed through pyramiding more salt-tolerance genes (loci) distributing on different *Thinopyrum* chromosomes.

## 3 Chromosomes (segments) regulating salt tolerance in *Thinopyrum* species

The salt tolerance in *Thinopyrum* is controlled by multiple genes which have been mapped on different chromosomes by using the wheat-*Thinopyrum* disomic addition and substitution lines. For instance, Dvořák et al. (1988) mapped salt tolerance of *Th. elongatum* on chromosomes 3E, 4E, and 7E. A field evaluation showed that all seven chromosomes except 6E of *Th. elongatum* enhanced salt tolerance in the CS substitution lines, and 3E had the largest effect (Omielan et al., 1991). Both 3E and 4E were confirmed to maintain high ratios of K^+^/Na^+^ in the presence of 200 mM NaCl (Gorham, 1994).

Due to the diversity of genetic background of the plant material and the inconsistent conditions of different experimental setups, the identified chromosomes associated with salt tolerance are not always repeatable. Evidence suggests that tolerance to salt shock and gradual salt stress is regulated by different chromosomes. Salt shock tolerance, induced by a sudden exposure to 250 mM NaCl, is primarily governed by chromosomes 3E and 5E, whereas gradual salt stress tolerance, induced by 50 mM NaCl increments every three days up to 250 mM, is predominantly regulated by 3E, 4E, and 5E. However, the ditelosomic analysis further revealed that salt shock tolerance is associated with 1EL, 5ES, 5EL, 6EL, 7ES, and 7EL, while gradual salt stress tolerance is linked with 1ES, 1EL, 5ES, 5EL, 6ES, 7ES, and 7EL (Zhong and Dvořák, 1995). Chromosome 5E enhances tolerance to 250 mM NaCl by stabilizing the photosystem II complex and cytochrome pathway (Kasai et al., 1998). The K^+^/Na^+^ ratios in *Th. elongatum* subjected to 100 and 250 mM NaCl stress were mapped to 1ES, 7ES, and 7EL (Deal et al., 1999). Acclimation to salt stress in *Th. elongatum* is mainly governed by chromosome 3E, which is regulated by abscisic acid (ABA) and is accompanied by elevated expression of salt-responsive genes (Noaman et al., 2002). A recombinant line, 524-568, incorporating the smallest *Th. elongatum* 3EL chromatin segment onto the distal end of wheat chromosome 3AL, was shown to be responsible for Na^+^ exclusion under salt stress (Mullan et al., 2009). Moreover, chromosome 5J (=5E^b^) of *Th. bessarabicum* harbors a major gene(s) for salt tolerance, characterized by Na^+^ exclusion from the leaves and roots (Forster et al., 1988; King et al., 1996; Mahmood and Quarrie, 1993).

*Th. distichum* possesses a genome constitution of J_1_^d^J_1_^d^J_2_^d^J_2_^d^. From the cross between the *Th. distichum*-*Secale cereal* (2n=4x=28) amphiploid and diploid rye, fifteen F_1_ plants with high levels of salt tolerance were identified (Marais and Marais, 2003). Chromosomes 2J_1_^d^, 3J_1_^d^, 4J_1_^d^, 5J_1_^d^, and 7J_1_^d^ were shown to be associated with salt tolerance in *Th. distichum* (Marais and Marais, 2003), with chromosome 3J_1_^d^ exerting the strongest effect, followed by 5J_1_^d^ and 7J_1_^d^ (Botes and Marais, 2007). The introduction of chromosomes 2J_1_^d^ and 3J_1_^d^ into triticale appears to be the only combination that significantly enhances survival rate under salt stress. Furthermore, the addition of 2J_1_^d^, 3J_1_^d^, and 5J_1_^d^ or of 3J_1_^d^, 4J_1_^d^, and 5J_1_^d^ resulted in salt tolerance comparable to that of the primary *Th. distichum*-triticale amphiploid (Marais et al., 2007).

The wheat-*Th. ponticum* 4E(4D) substitution line exhibited a higher K^+^/Na^+^ ratio than common wheat under 150 mM NaCl stress (Xu et al., 1998). In the salt-tolerant cultivar Shanrong 3, salt tolerance has been associated with the SSR marker *Xgwm 304* on wheat chromosome 5A (Shan et al., 2006). The wheat addition line AJDAj5 displayed salt tolerance comparable to that of wheat-*Th. junceum* partial amphiploids, and several salt-tolerant recombinant lines were subsequently developed from AJDAj5 (Wang et al., 2003). More recently, Zeng et al. (2023) developed a novel 3E(3D) substitution line derived from wheat × tetraploid *Th. elongatum*, which exhibited a lower Na^+^ concentration and higher K^+^/Na^+^ ratio in both shoots and roots under salinity stress compared with its wheat parents. This line also demonstrated a higher photosynthesis rate, improved water-use efficiency, greater antioxidant capacity, and enhanced osmotic adjustment under salt stress. Collectively, chromosomes from homologous groups 3 and 5 appear to play a key role in the regulation of salt tolerance in *Thinopyrum* species. Further research is required to delimit salt tolerance genes to small genome intervals to facilitate their utilization in cereal and forage crop improvement.

## 4 Salt-responsive genes in *Thinopyrum* species

Salt tolerance in *Th. elongatum* is closely correlated with the expression levels of the salt-responsive genes. A few early salt-induced (*ESI*) genes were markedly induced in roots of *Th. elongatum* within 2 h and peaked after 6 h exposure to 250 mM NaCl (Gulick and Dvořák, 1992; Galvez et al., 1993). Eleven *ESI*s were mapped on *Th. elongatum* chromosomes, of which *ESI4*, *ESI14*, *ESI15*, *ESI28*, and *ESI32* were located in homoeologous group 5, *ESI18* and *ESI35* in group 6, and *ESI47*, *ESI48*, *ESI3*, and *ESI2* in groups 1, 3, 4, and 7, respectively (Dubcovsky et al., 1994). The Salt Overly Sensitive (SOS) pathway plays a central role in plant salinity tolerance through modulation of the SOS core proteins at the transcriptional and post-translational levels (Ali et al., 2023). Among the *Arabidopsis*-rice-wheat gene orthologues for Na^+^ transport genes, *SOS1* were mapped on 1EL and 3ES, *NHX5* on 5EL, *AVP2* on 6ES, *AVP1* on 7ES, and *HKT1* on 7EL, respectively (Mullan et al., 2007). The salt-responsive genes expressed differentially between the salt-tolerant and salt-sensitive *Th. ponticum* ecotypes under salt stress. For instance, the salt-tolerant lines showed higher expression of *HKT1;4* and *NAC9* in the leaves and roots than the salt sensitive lines (Sheikh-Mohamadi et al., 2022). In addition, the expression levels of *NHX7.1/SOS1* and *NCL1* were higher in the salt-tolerant *Th. ponticum* lines than in the salt-sensitive lines (Xiao et al., 2025).

Transcriptomic analysis can reveal more salt-response genes and discover novel mechanisms of salt tolerance in halophytes (Meng et al., 2018; Fan, 2020; Feng et al., 2025). In *Th. ponticum* exposed to 150 mM NaHCO_3_, 1833 and 1536 differentially expressed genes (DEGs) were identified in leaves and roots, respectively. Functional enrichment highlighted pathways involved in antioxidant biosynthesis, ion binding, and phenylalanine/phenylpropanoid metabolism. Enriched ion binding categories featured *BAK1*, *CIPK10*, *STRK1*, *WAK8*, and multiple laccase genes (Zhang et al., 2025a). Under 150 mM Na_2_SO_4_, 1682 leaf DEGs and 2816 root DEGs were identified, primarily associated with redox homeostasis, ion homeostasis, and signal transduction. Collectively, *Th. ponticum* appears to coordinate NAC/MYB/WRKY transcription factors, salicylic acid and ethylene signaling, and Ca^2+^-dependent pathways to cope with salt stress. Nine candidate genes, including *UGT7472*, *JMT*, *T4E14.7*, *CAX5*, *CP1*, *PXG2*, *NAMT1*, *BON3*, and *APX7*, have been proposed to contribute to salt stress in *Th. ponticum* (Zhang et al., 2025b).

Transcriptomic investigations of wheat-*Thinopyrum* materials have largely focused on amphiploids, substitution lines, and introgression lines. Across comparisons among the common wheat cultivar ‘Chinese Spring’ (CS), CS-*Th. elongatum* amphiploid, and the 3E(3A) substitution line, 304 DEGs were detected, 18 of which are involved in signal transduction or regulatory function. These include transcription factors, protein kinases, ubiquitin ligases, and genes participating in phospholipid signaling (Hussein et al., 2014). In the CS-*Th. elongatum* amphiploid Y1805, transcriptional regulatory networks respond both to salt stress and the subsequent recovery phase. Salt-responsive DEGs are enriched for peroxisomal processes, arginine and proline metabolism, starch and sucrose metabolism, chlorophyll and porphyrin metabolism, and photosynthesis (Peng et al., 2022). Furthermore, assay for transposase-accessible chromatin using sequencing (ATAC-seq) profiling of Y1805 identified 85 motifs within 1776 location-specific peaks and 478 motifs within peaks with altered accessibility under salt stress. Motif-associated transcription factors are dominated by the MYB family, followed by AP2/EREBP, bZIP, bHLH, and WRKY families. Gene Ontology (GO) analyses integrating ATAC-seq accessibility and RNA-seq expression indicate significant enrichment of organic and carboxylic acid catabolic process, cellular hormone and cytokinin metabolic, and cellular amino acid catabolism in Y1805 (Tian et al., 2024). These findings from wheat-*Thinopyrum* backgrounds provide a comparative framework for interpreting salt-response mechanisms in *Th. ponticum*.

Transcriptomic analyses of the CS-*Th. elongatum* amphiploid Y1805 indicates that several *Th. elongatum* salt-responsive genes, including *TtLEA2-1* (*Tel3E01G270600*; Yang et al., 2022), *TtWRKY256* (*Tel1E01T143800*; Li et al., 2022), *TtERF_B2-50* (*Tel2E01T236300*; Liu et al., 2023), *TtNAC477* (*Tel3E01T644900;*Liu et al., 2024), *TtMYB1* (*Tel2E01G633100*; Mu et al., 2024), and *TtbHLH310* (*Tel1E01T336100*; Li et al., 2025), show high expression across roots, stems, and leaves under salt stress. Functionally, overexpression of *TtMYB1* (Mu et al., 2024) and *TtHSF6-1* (*Tel7E01G472900*; Tian et al., 2024) enhanced salt tolerance in wheat, supporting their utility for genetic improvement. A root-specific vacuolar Na^+^/H^+^ antiporter gene, *AeNHX1*, was isolated from *Th. elongatum*, its overexpression conferred increased salt tolerance in *Arabidopsis* and *Festuca* species (Qiao et al., 2007). Consistent with the role of ion transport, exclusion of Na^+^ from leaves and vacuolar or cell-type-specific compartmentation depend on changes in ion transporters expression (Munns and Tester, 2008). From *Th. ponticum*, a salt-induced high-affinity potassium transporter, *EeHKT1;4*, was cloned (Zhang et al., 2022b); its overexpression in *Arabidopsis* improved tolerance to salt and drought and reduced reactive oxygen species under stress (Zhang et al., 2022b).

Beyond *Thinopyrum*, transcriptome-guided analyses identified a putative potassium channel gene implicated in tissue-level salt tolerance in wheat recombinant lines W4909 and W4910 derived from AJDAj5 × Ph^I^ (bearing the *Ph^I^* allele from *Aegilops* sp*eltoides*) (Mott and Richard, 2007). Comparative profiling further showed higher expression of genes involved in stress response, unsaturated-fatty-acid and flavonoid biosynthesis, and the pentose-phosphate pathway in Shanrong 3 versus Jinan 177 (Liu et al., 2012). Under alkaline stress, 5691 and 5932 long noncoding RNAs (lncRNAs) were identified in Shanrong 4 and Jinan 177, respectively (Wei et al., 2022), suggesting that differential lncRNAs-mediated regulation of alkaline tolerance between these genotypes.

## 5 Differentially expressed proteins responding to salt in *Thinopyrum* species

Proteomic studies indicate that a broad set of mitochondrial proteins responds to salinity in CS and CS-*Th. elongatum* amphiploid, notably enzymes involved in detoxifying reactive oxygen species (ROS), including manganese superoxide dismutase, serine hydroxymethyltransferase, aconitase, malate dehydrogenase, and β-cyanoalanine synthase (Jacoby et al., 2013). In total, 44 and 102 differentially expressed proteins (DEPs) were identified in Y1805 under salt stress and recovery process, respectively (Yang et al., 2021), among which eight DEPs were specifically responsive to salt stress. Relative to CS, pathways in Y1805 were characterized by energy and lipid metabolism during recovery, whereas antioxidant activity and molecular function regulator activity were prominent under salt stress; during recovery, the GO terms “virion” and “virion part” were also recorded. In seedling roots, 114 DEPs distinguished Shanrong 3 from Jinan 177 under salt stress, spanning signal transduction, transcription/translation, transport, chaperones, proteolysis, and detoxification etc (Wang et al., 2008). Across leaves and roots, 93 and 65 DEPs were detected under drought and salt stress, respectively (Peng et al., 2009). Shanrong 3’s salt tolerance has been linked to more efficient removal of toxic by-products, supported by stronger osmotic and ionic homeostasis and better post-stress regrowth (Peng et al., 2009).

## 6 Mechanism of salt tolerance in *Thinopyrum* species

The mechanisms of salt-alkali tolerance in *Th. ponticum* have been reviewed (Andrioli, 2023), and primarily encompasses Na^+^ exclusion, high K^+^/Na^+^selectivity, and osmotic adjustment (osmoprotection). Forage productivity of *Th. ponticum* correlates with leaf Na^+^ exclusion and the K^+^/Na^+^ ratio under saline conditions (Johnson, 1991). Salinity tolerance has also been associated with Na^+^ and Cl^−^ concentrations in fodder (Bhuiyan et al., 2017) and with greater uptake and selective transport of K^+^ over Na^+^ (Guo et al., 2015). Additional contributing traits include elevated proline and soluble sugars (Shannon, 1978); high water-use efficiency with relatively invariant ^13^C discrimination (Johnson, 1991); and maintenance of shoot osmolality via regulation of Na^+^ and K^+^ contents, while mitigating Ca^2+^ deficiency during salt stress (Zhang et al., 2005).

Similarly, increased salt tolerance of wheat-*Thinopyrum* amphiploid correlates with Na^+^ and Cl^−^ exclusion, K^+^ retention, and K^+^ retranslocation within shoots (Gorham et al., 1986b; Omielan et al., 1991; Schachtman et al., 1989; Santa-María and Epstein, 2001). In the CS-*Th. elongatum* amphiploid, low Na^+^, high K^+^, and accumulation of glycine betaine in young leaves were associated with enhanced salt tolerance (Colmer et al., 1995). By contrast, the CS-*Th. bessarabicum* amphidiploid did not inherit the high glycine betaine concentrations characteristic of *Th. bessarabicum* (Gorham et al., 1986b). In Y1805, salt tolerance has been linked to strengthened cell walls, reactive oxygen species scavenging, osmoregulation, phytohormone regulation, transient growth arrest, enhanced respiration, transcriptional regulation, and error information processing (Yang et al., 2021). The wheat-*Th. ponticum* introgression line Shanrong 3 exhibits higher selectivity for K^+^ over Na^+^, thereby limiting Na^+^ transport from root to shoot (Shan et al., 2008).

**Figure 1:**
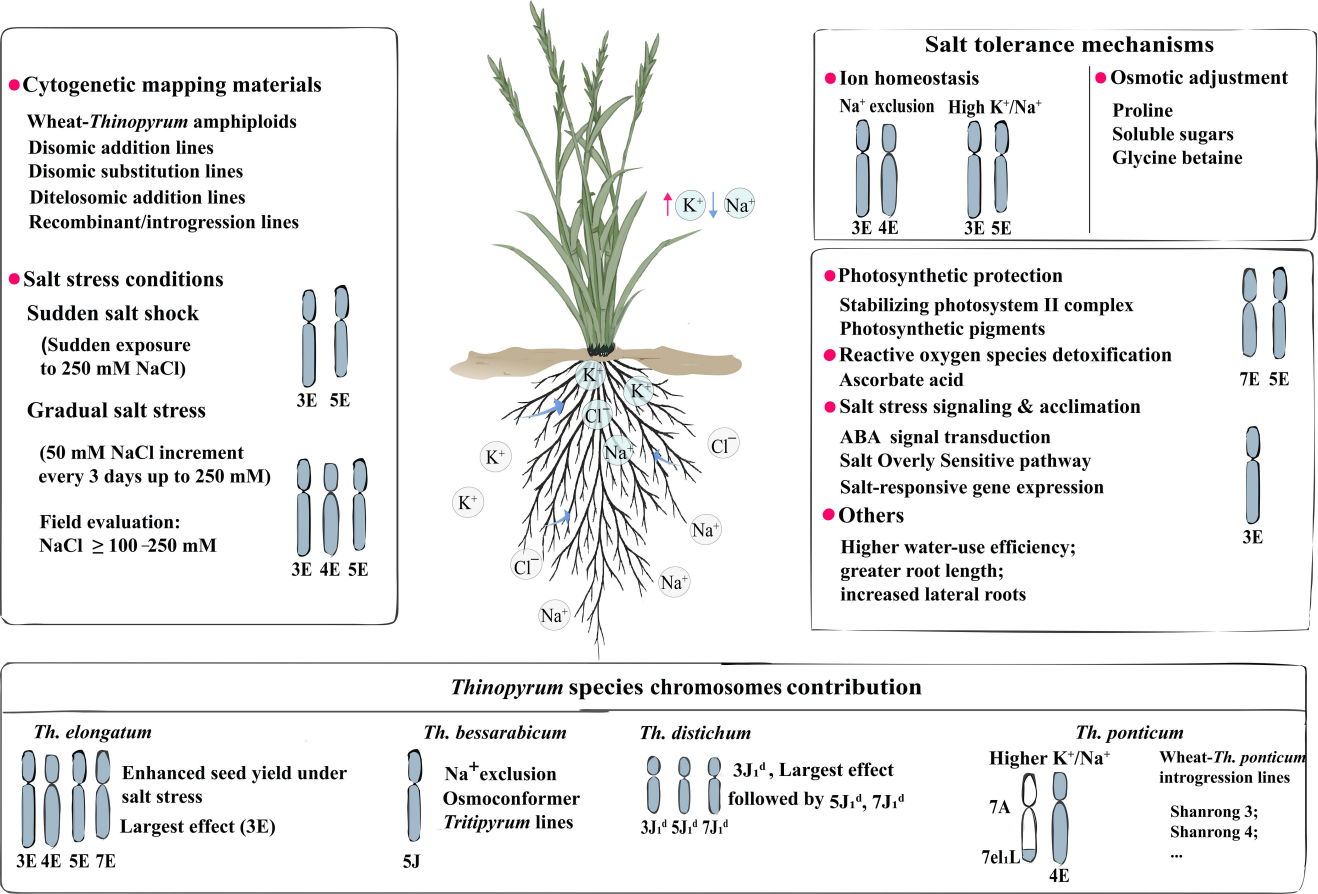
Mechanisms and chromosomes (segments) regulating salt tolerance in *Thinopyrum* species. The upper two boxes were principally summarized from *Th. elongatum*.

Although *Th. ponticum* is widely regarded as highly salt-tolerant, intraspecific variation and its mechanisms remain underexplored (McGuire and Dvoák, 1981; Shannon, 1978; Borrajo et al., 2021, 2022). Phenotyping indicates that salt-tolerant ecotypes exhibit greater initial root length and lateral root density, together with lower accumulation of reactive oxygen species and malondialdehyde (MDA), than the salt-sensitive ecotypes (Sheikh-Mohamadi et al., 2022). High-resolution omics, including single-cell RNA sequencing, and spatial transcriptomics help discover novel mechanisms of salt tolerance in *Thinopyrum* species. The mechanisms and chromosomes (segments) regulating salt tolerance in *Thinopyrum* species were summarized in **Figure 1**.

## 7 Prospect for exploiting salt tolerance in *Thinopyrum* species for forage and food production

Although *Th. ponticum* has substantial potential as a saline pasture, a bioenergy crop, and for soil reclamation on marginal lands including saline-alkaline soils (Falasca et al., 2017; Csete et al., 2011; Ciria et al., 2020; Scordia et al., 2022), it remains underutilized due to poor management and limited attention (Smith, 1996). In China, it has long served as a wild donor for distant hybridization in wheat, but has been used less as a forage crop since its introduction in the 1950s. Recently, a “Coastal Grass Belt” has been proposed for coastal saline-alkaline soils around the Bohai Sea that are unprofitable for food crops. Such a belt would help meet the growing demand for high-quality forage while minimizing competition with food crops for arable land (Xu et al., 2022; Wang et al., 2022; Li et al., 2023b). The deployment of *Th. ponticum* varieties that combine enhanced salt and alkali tolerance with high productivity will largely determine the scale of utilization. A comprehensive and standardized evaluation of salt tolerance across *Thinopyrum* species is needed. Molecular breeding strategies, including development of polymorphic molecular markers, construction of high-density linkage maps, genome-wide association studies (GWAS), pyramiding of salt tolerance genes, and genome editing, will accelerate the breeding of *Th. ponticum* varieties. The growing genome sequences of *Thinopyrum* species enable researches to map and isolate salt tolerance genes in *Thinopyrum* species. For instance, Wang et al. (2020a) reported for the first time the genome sequence of *Th. elongatum* (acc. D-3458) which has a 4.63 Gb assembly. In addition, two genome sequences of *Th intermedium* v2.1 with 10.92 Gb of assembled sequence (Thinopyrum intermedium v2.1 DOE-JGI) (Thinopyrum intermedium v2.1 DOE-JGI), and *Th. intermedium* (acc. PI 440031) with 10.89 Gb of assembled sequence (Sun et al., 2025) were also available. F_1_ progeny from wheat × *Th. ponticum* crosses retain the perennial habit of *Th. ponticum* and can grow faster and produce more forage than *Th. ponticum* itself (Wang et al., 2020b), suggesting a new avenue for forage production if clonal seed production can be enabled through genome editing. In addition, salt-tolerant forage crops such as *Tritipyrum* and perennial wheat may be developed from crosses between wheat or rye and *Thinopyrum* species (Hassani et al., 2000). Several salt-tolerant *Tritipyrum* lines with high straw and grain yields have been reported (Kamyab et al., 2012, 2017, 2018). Salt-tolerant wheat introgression lines carrying small *Thinopyrum* chromosome segments without evident linkage drag, such as Shanrong 3 and Shanrong 4, can be deployed directly as wheat cultivars. Furthermore, salt-tolerance genes isolated from *Thinopyrum* species may serve as molecular targets for improving other crops, including wheat.

## 8 Conclusions

Species in the genus *Thinopyrum* exhibit high levels of tolerance to salt and alkali stress, and this trait appears polygenic with largely additive effects. Progeny from wheat × *Thinopyrum* crosses with enhanced salt tolerance can be used as both forage and grain crops. Using chromosome addition and substitution lines, salt tolerance loci have been mapped to multiple chromosomes of *Th. elongatum*, *Th. bessarabicum*, *Th. distichum*, and *Th. ponticum*, with homologous group 3 and group 5 showing the largest effects. Transcriptomic and proteomic analyses have identified many salt-responsive genes (proteins), yet only a small subset has undergone functional validation. Going forward, mechanisms of salt tolerance and the associated transcription regulatory networks should be resolved through comprehensive and standardized phenotyping across *Thinopyrum* germplasm together with genome-wide association studies (GWAS) and targeted functional studies.
